# Evaluating lumbar disc degeneration by MRI quantitative metabolic indicators: the perspective of factor analysis

**DOI:** 10.1186/s13018-024-04726-8

**Published:** 2024-05-06

**Authors:** Boxin Zheng, Lin Ouyang, Jianhua Shi, Xiaochan Shen, Hanbin Lei

**Affiliations:** 1 Radiology Department, 909th Hospital (Affiliated Southeast Hospital, Xiamen University), Zhangzhou, China; 2 School of Mathematics and Statistics, Minnan Normal University, Zhangzhou, China; 3 Institute of Medical Imaging Medical College, Xiamen University, Xiamen, China; 4 Fujian Key Laboratory of Granular Computing and Applications, Zhangzhou, China

**Keywords:** Lumbar disc degeneration, Quantitative indicators of biological metabolism, Factor analysis method

## Abstract

**Purpose:**

This study aimed to investigate an early diagnostic method for lumbar disc degeneration (LDD) and improve its diagnostic accuracy.

**Methods:**

Quantitative biomarkers of the lumbar body (LB) and lumbar discs (LDs) were obtained using nuclear magnetic resonance (NMR) detection technology. The diagnostic weights of each biological metabolism indicator were screened using the factor analysis method.

**Results:**

Through factor analysis, common factors such as the LB fat fraction, fat content, and T2* value of LDs were identified as covariates for the diagnostic model for the evaluation of LDD. This model can optimize the accuracy and reliability of LDD diagnosis.

**Conclusion:**

The application of biomarker quantification methods based on NMR detection technology combined with factor analysis provides an effective means for the early diagnosis of LDD, thereby improving diagnostic accuracy and reliability.

## Introduction

The intervertebral disc (ID), located between two vertebral bodies, is a dynamic fluid structure composed of three parts: the nucleus pulposus (NP), annulus pulposus (AF), and cartilage endplate (CEP) [1, 2]. The nucleus pulposus is located in the central region and is rich in proteoglycans (PGs), which maintain the water content and height of the ID. Its main function is to buffer the axial load of the spine [3, 4]. The AF is composed of organic collagen fibres and some fibroblast-like cells [5, 6], and it supports the gel-like structure of the NP by wrapping the ID in a layered structure to resist the external force imposed on ID components [7]. The CEP is two transparent cartilages located on the upper and lower sides of the ID that are directly connected to the vertebral bone tissue [5]. As an individual ages, the IDs undergo a degradation cycle that begins earlier and progresses faster than other tissues in the body [8]. Therefore, the ID is one of the most sensitive organs in the ageing process of the human body and can reflect changes in human function.

Lumbar disc degeneration (LDD) is the pathological and physiological process in which significant changes occur in the shape, structure, and physiological function of the lumbar disc (LD), ultimately leading to a decrease in its ability to withstand pressure and torsional load. The pathogenesis of LDD is complex and closely related to age, genetics, excessive mechanical loading, trauma, and other factors. LDD is an important cause of low back pain in adults, and it is also the pathological and physiological basis for degenerative diseases such as lumbar spinal stenosis and lumbar disc herniation. In severe cases, it can even lead to the loss of a patient’s ability to work. If the signs of LDD can be detected in a timely manner at an early stage, early intervention and treatment can be carried out, which can effectively prevent the escalation of the lesion level and avoid the occurrence of irreversible consequences. Pfirrmann et al. [9] first proposed a method based on morphological and structural MRI indicators to determine the severity of LDD termed the Pfirrmann grading method. This method divides LDD into five progressive regression levels, I to V, and is recognized as the gold standard for determining the degree of LDD. With these criteria, doctors can determine the severity of LDD in a patient based on indicators such as morphological changes in the LD and the degree of MRI signal reduction. These MRI techniques often rely on the experience of diagnostic doctors to determine the severity of LDD. However, in patients with unclear symptoms of early LDD, the accurate detection of LDD using traditional diagnostic methods is difficult.

Previous studies have shown that abnormal tissue metabolism occurs earlier than changes in tissue structure. In the early stages of LDD, the biochemical metabolism of the LB and LDs changes [10, 11]. These changes can be used to assist in earlier LDD evaluation, improve diagnostic accuracy, and provide more accurate treatment. To address this issue, this article used a technical method based on quantitative detection of biochemical metabolites by MRI to observe characteristic metabolic changes during LDD.

## Materials and methods

### Study population

This study was approved by the Medical Ethics Committee of the 909th Hospital in China (Southeast Hospital Affiliated to Xiamen University, China), and informed consent was obtained from the study subjects. From June 2022 to December 2022, 99 volunteers of different age groups (20–29 years, 30–39 years, 40–49 years, 50–59 years, 60–69 years, and 70–79 years) were recruited. There were a total of 48 males (average age 45.9 ± 13.7 years) and 51 females (average age 48.8 ± 9.8 years), with a body mass index (BMI) of 23.1 ± 2.7 kg/m${^2}$ (range 16.71$-$30.86 kg/m${^2}$).

The inclusion criteria were as follows: (1) normal socialization with the general population, normal language communication, and ability to cooperate with MRI; (2) healthy with no major illnesses and voluntary participation; (3) age 20–80 years; and (4) no differences in sex.

The exclusion criteria were as follows: (1) contraindications for MRI examination, including claustrophobia or magnetic metal implants in important organs, such as cardiovascular and cerebrovascular stents; (2) normal structure and metabolism of the lumbar spine altered by other factors, such as deformities, lumbar fractures, surgical history, infections, connective tissue diseases, endocrine diseases, tumours, and long-term medication; (3) behaviours that may affect lumbar metabolism within one day prior to MRI examination, including medication (such as medications promoting blood circulation and resolving stasis, vasodilators, and stimulants), alcoholism, physical therapy (such as lumbar microwave, radio-frequency, or massage), etc.; and (4) implants that affect the quality of MRI imaging, including giant metal grafts, such as artificial hip joints and pelvic internal fixation.

A total of 32 volunteers met the criteria and were included in the data analysis. The participants were aged 25–71 years, including 14 males, with an average age of 48.7 ± 12.6 years, and 18 females, with an average age of 47.6 ± 11.1 years, as detailed in Tables 1 and 2.

**Table 1:** Basic information

	Features	Number (percentage)
Sex	Male	14 (44)
	Female	18 (56)
	BMI (kg$/m^2$)	22.4
	LD ($L_{3/4},L_{4/5},L_5/S_1$)	96
	Vertebral body ($L_{3},L_{4},L_5$)	96
Pfirrmann grade	I	8 (8)
	II	10 (10)
	III	26 (27)
	IV	23 (24)
	V	29 (30)
Age (years)	20$\sim$29	4 (13)
	30$\sim$39	3 (9)
	40$\sim$49	9 (28)
	50$\sim$59	12 (38)
	60$\sim$69	3 (9)
	70$\sim$79	1 (3)

**Table 2:** Overview of Pfirrmann grades for L$_{3/4}$, L$_{4/5}$, and L$_5/$S$_1$ (unit: numbers)

Pfirrmann grade	L$_{3/4}$	L$_{4/5}$	L$_5/$S$_1$	Total
I	2	4	2	8
II	7	1	2	10
III	8	11	7	26
IV	8	8	7	23
V	7	8	14	29
Total	32	32	32	96

### Method

**Fig. 1:**
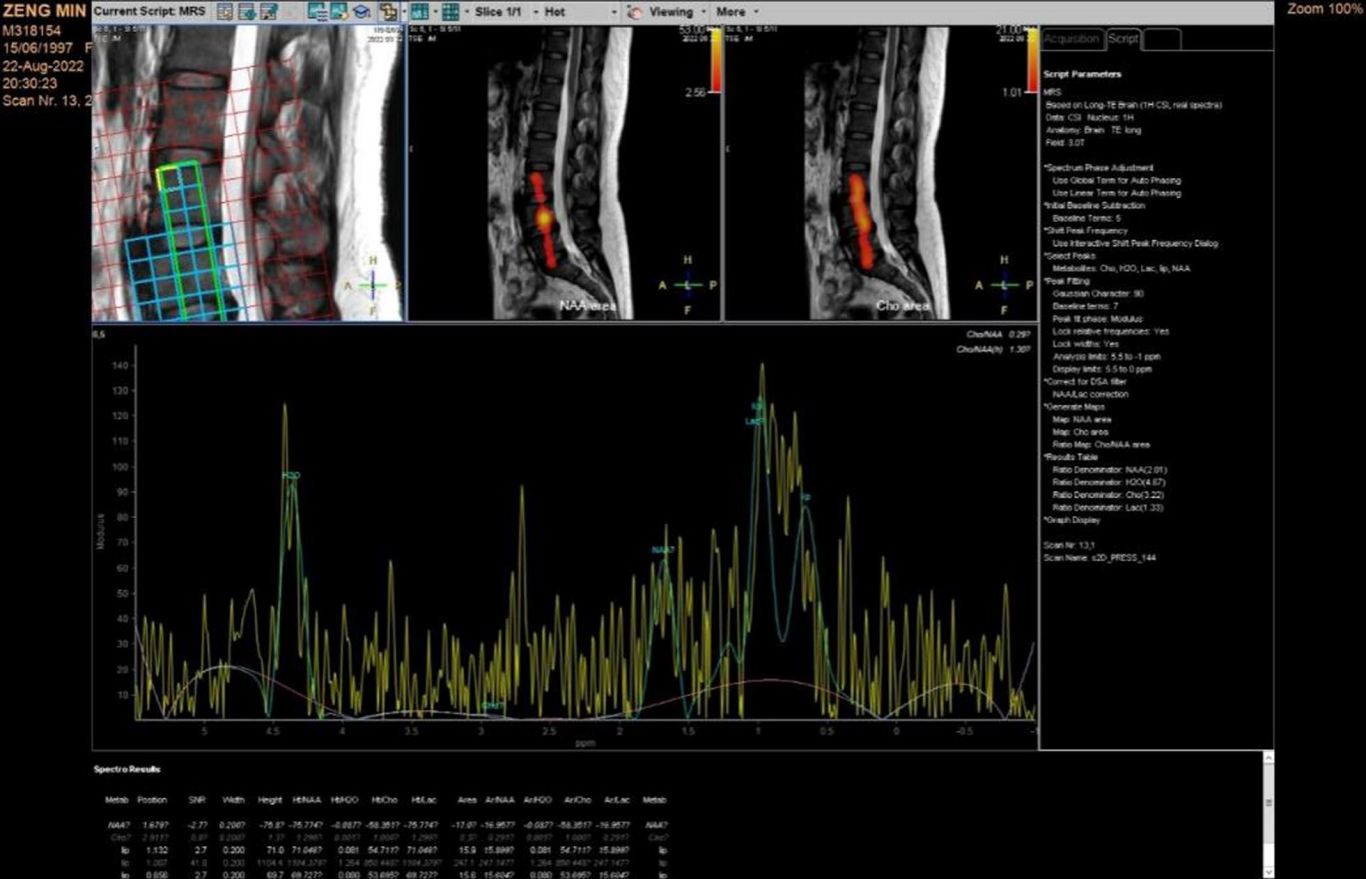
Measurement of N-acetyl, Lip, H$_2$O, Lac, and Cho metabolites in the ROI

**Fig. 2:**
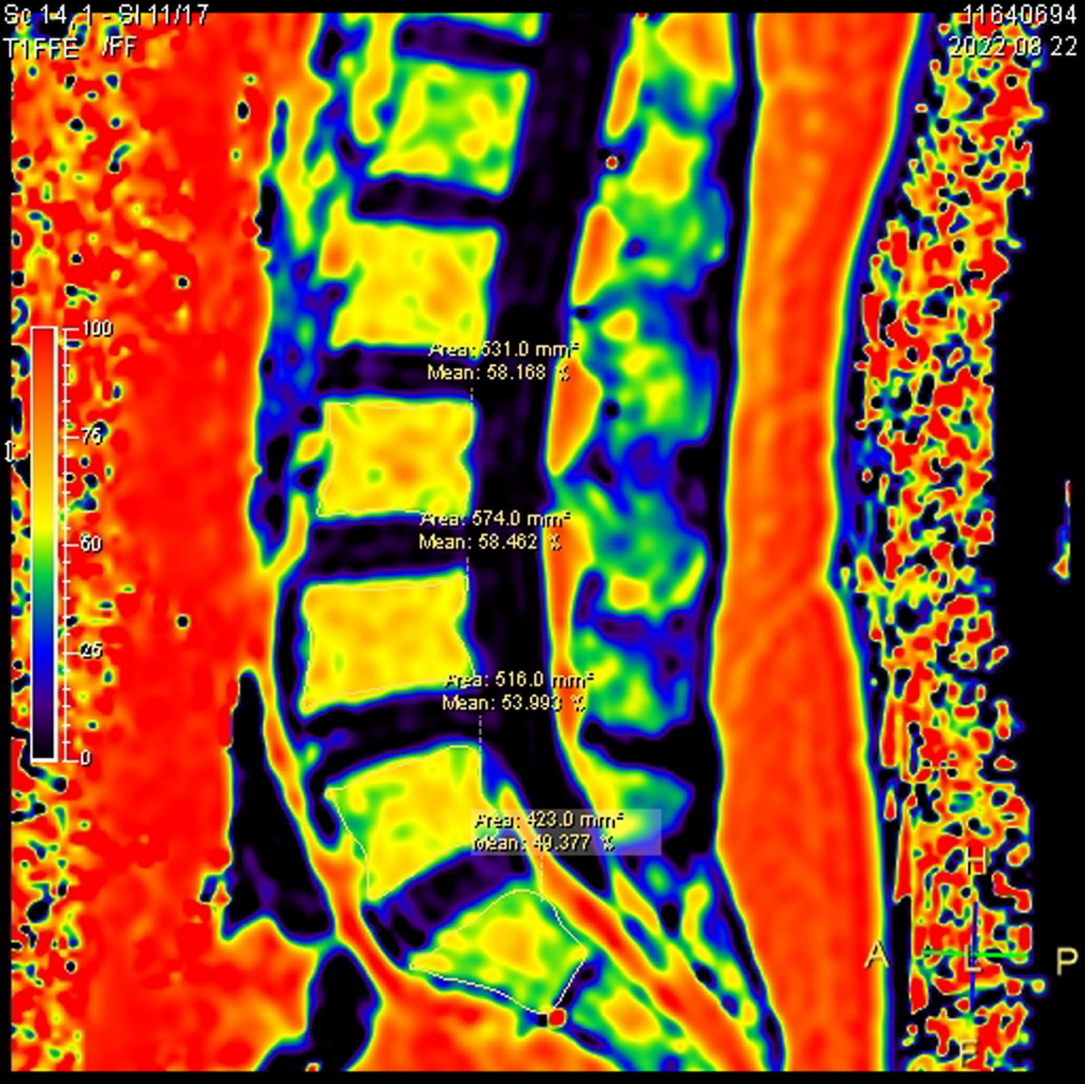
Measurement of the lumbar FF value of the vertebral body

**Fig. 3:**
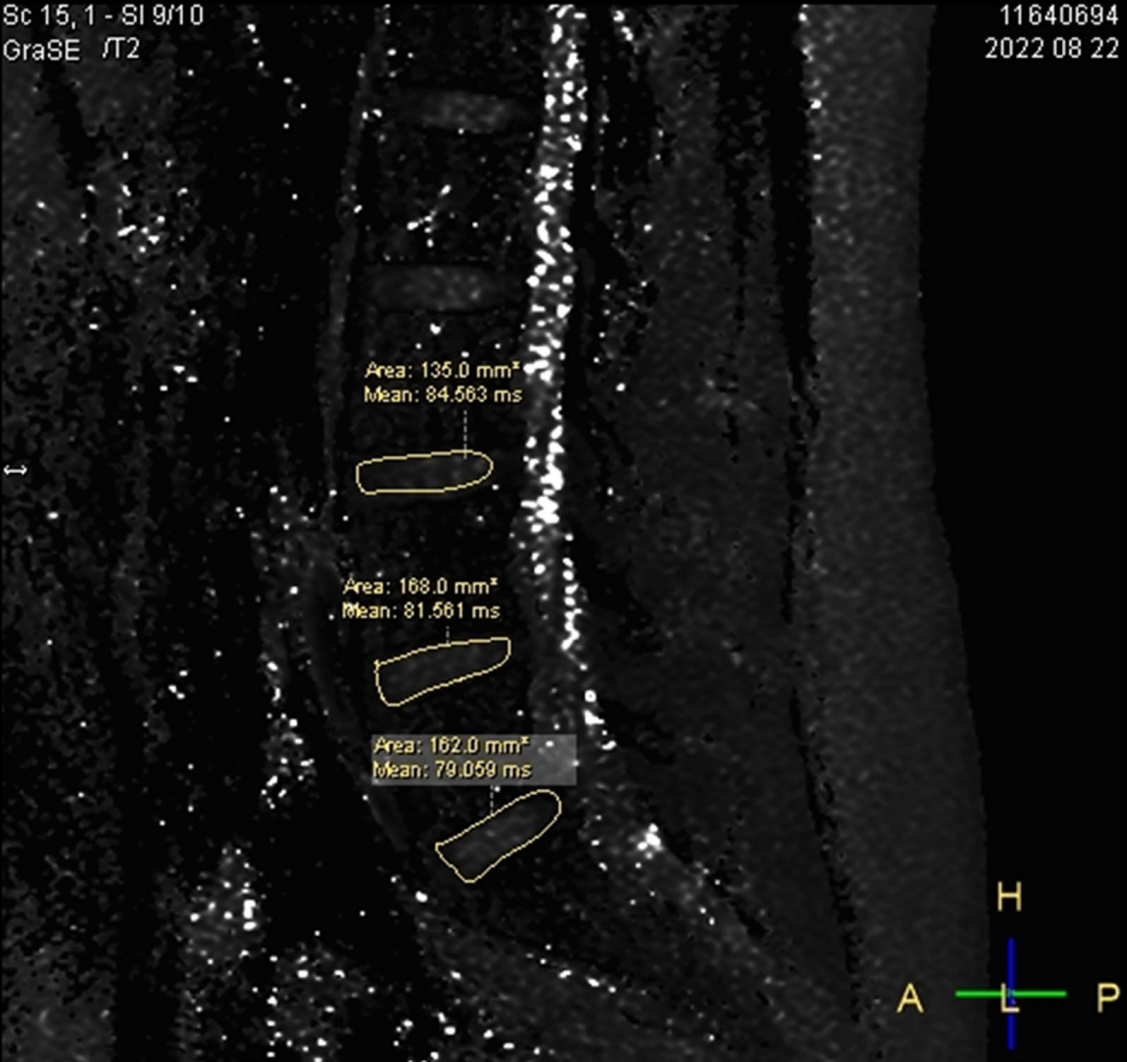
T2* measurement of LDs

1. Testing equipment: A 3.0T MR scanner (Philips Ingenia 3.0T, Netherlands) with a 32-channel coil on the spinal surface was used to measure metabolic indicators.
2. Research site: The lower lumbar spine bears more human weight than the upper lumbar spine, and the LBs and LDs are relatively more affected by endogenous heavy stress disturbances. Therefore, we chose the L$_3$, L$_4$, and L$_5$ LBs and the L$_{3/4}$, L$_{3/4}$, and L$_5/$S$_1$ LDs as the observation targets.
3. Region of interest (ROI): The midsagittal plane of the lumbar spine was selected as the observation plane to better observe the structure of the LBs and LDs. The LB medullary region and LD nucleus pulposus region were selected as the ROIs for measurement. The delineation area along the inner edge of the target LB bone cortex was manually drawn as the medullary ROI (avoiding the upper and lower endplates and bone cortex areas), and the delineation area along the inner edge of the target LD fibre ring was manually drawn as the ROI for the NP.
4. Detection technology
  1. Magnetic resonance spectroscopy (MRS) was used for quantitative analysis of the biochemical metabolites of the LBs and LDs and extraction of information such as the area under the curve of relevant evaluation indicators.
  2. MRI m-DIXON Quant imaging was used to measure the FF value of tissue within the ROI, reflecting the proportion of fat tissue volume within the ROI. The measurement process was repeated three times to reduce errors, and the average value was taken as the final evaluation value.
  3. MRI T2* imaging was used to measure the T2* value of the tissue within the ROI, reflecting the hydration state of the tissue. The measurement process was repeated three times to reduce errors, and the average value was taken as the final evaluation value.
5. The detection indicators included MRI morphological and structural indicators and quantitative indicators of biological metabolism. MRI quantitative indicators were obtained using MRI technology, usually manifested as specific quantitative values or parameters, and are often widely used in clinical medical fields such as imaging disease diagnosis and treatment monitoring.
  1. Morphological structure indicators represent the anatomical structure and pathological changes in tissues and organs. In this study, a Pfirrmann grading system, which is a method for evaluating the degree of LD degeneration based on the strength, structure, height, and endplate shape of LD signals, was used. The scoring system is divided into five levels, with Level I indicating normal LDs and Level V indicating severe degenerative changes. The Pfirrmann scoring criteria for LD are shown in Table 3.
  2. Biometabolic indicators reflect the relative content of biochemical metabolites in the body’s tissues. In this study, the indicators included MRS detection of N-acetyl, lipids, H2O, choline, and lactic acid (Lac) in the LBs. The values are usually measured using the area under the peak of the substance spectrum curve, such as A$_{H_2O}$, as well as MRI m-DIXON Quant measurements of the fat fraction (FF) and T2* imaging measurements of the LD T2* value (Figs. 1, 2, 3). A$_{H_2O}$: The A$_{H_2O}$ spectrum at a chemical shift of 4.8 ppm in MRS can be used to evaluate the changes in water content in vertebral tissue by measuring the area under the peak of the curve spectrum. A$_{N-acetyl}$: The spectrum of N-acetyl, one of the metabolites of PGs, at a chemical shift of 2.23 ppm in MRS reflects the changes in intervertebral disc decomposition. $\text{A}_{Lip}$: The sum of the lip peak areas at 1.24 ppm, 1.12 ppm, and 0.8 ppm for MRS chemical shifts mainly reflects the methyl-(-CH3) and vinyl(-CH2-)-containing substances in adipose tissue in the ROI, which can help assess the relative content of adipose tissue in the vertebral body. A$_{Lac}$: The lac spectrum at a chemical shift of 1.3 ppm in MRS can be used to evaluate changes in oxygen metabolism in vertebral tissue by measuring its area below the peak. FF: Analysing the proportion of fat volume in the ROI, reflecting the distribution of LB fat, can provide important information for determining LDD grade. The FF of the adjacent upper and lower vertebrae of the intervertebral disc is represented by FF$_U$ and FF$_L$, respectively. T2* value: Reflects the macroscopic content of $H_2O$ in LDs, expressed as the T2* value, which is closely related to the structural integrity of the fibre ring.
6. Image processing: After inputting the original images and data into the Intelligent Portal Workstation (Philips), two radiologists who have 10 years and 8 years of experience in MR diagnosis and who specialize in bone and joint disease research performed double-blind postprocessing analysis and judgement on the images, including measurements of MRS postprocessing, the FF value of the LBs, and the T2* value of the LDs.

**Table 3:** Characteristics of each degeneration grade

Grade	Structure	Boundary	Signal strength	LD height
I	Uniform, white	Clear	High signal	Normal
II	Uneven, with or without clear and high signal stratification	Clear	High signal	Normal
III	Uneven, grey	Unclear	Moderate signal	Normal to mild reduction
IV	Unevenness, greyish black	Disappearance	Moderate low signal	Normal to moderate decrease
V	Unevenness, black	Disappearance	Low signal	Intervertebral space collapse

Boundary: The boundary between the nucleus pulposus and annulus fibrosus.

Signal: Signals such as cerebrospinal fluid

MRS postprocessing was performed using postprocessing software provided by the Philips magnetic resonance scanner workstation to perform spectral analysis on the images. Manual and automatic debugging of filtering, noise reduction, zero filling, and baseline phase correction steps were used to fit the data into standard spectral lines. The peak shape, peak value, area under the peak, and signal-to-noise ratio (SNR) of the wave were recorded. If the baseline in the spectrum was unstable, the full width at half height was too large, and the SNR $\le$ 2, the spectrum was considered unqualified. This study mainly measured A$_{N-acetyl}$, A$_{Lip}$, A$_{H_2O}$, and A$_{Lac}$ in the LB of interest. Due to the lack of vertebral display of A$_{N-acetyl}$ and, these two metabolic indicators were not analysed.

(7) Data analysis method: The factor analysis method was used to evaluate lumbar disc degeneration based on quantitative MRI metabolic indicators.

## Results

### MRI biochemical metabolic measurement indicators

A total of 96 samples were obtained and graded according to the LDD severity. The FF, A$_{Lip}$, A$_{Lac}$, and A$_{H_2O}$ of 96 LBs were measured in 32 patients, and the T2* values of 96 LDs were measured as quantitative MRI indicators in the same sample, as shown in Table 4.

**Table 4:** Basic information on biological metabolism

MRI quantitative indicator	Mean	SD	Number
T2*	63.23154	22.49313	96
FF$_U$	61.10629	12.14744	96
FF$_L$	63.84602	11.85228	96
Lip1.24	134.9217	96.21249	96
Lip1.12	194.6875	160.1563	96
Lip0.8	153.7731	141.1909	96
H$_2$O	1485.418	820.9532	96
Lac	387.8246	426.9371	96

#### Data preprocessing results

Factor analysis was used to reduce the dimensionality of the data. To eliminate the adverse effects caused by different dimensions, the original data were standardized, and KMO and Bartlett spherical tests were performed to verify whether the data were suitable for principal component analysis.

The measure of sampling adequacy for KMO sampling was 0.718, greater than 0.7, and the significance of Bartlett’s sphericity test was less than 0.01, indicating that the data were suitable for factor analysis (Table 5).

**Table 5:** KMO and Bartlett’s tests

Kaiser–Meyer–Olkin(KMO)	Measure of sampling adequacy	0.718
Bartlett’s test of sphericity	Approx. Chi-square	429.422
	df	28
	Sig.	0

#### Factor load matrix estimation and factor rotation

Table 6 shows the correlation coefficient matrix of various MRI quantitative indicators, with values closer to 1 indicating a stronger positive correlation and values closer to -1 indicating a stronger negative correlation. As shown in the table, there was a strong correlation between the FF values of adjacent vertebral bodies in the LDs. The T2* of LDs had a positive correlation with the A$_{H_2O}$ of adjacent vertebral bodies and a negative correlation with other quantitative MRI indicators. In addition, there was a positive correlation between the A$_{Lip}$ and A$_{Lac}$ of the LB. The determination of these correlations has guiding significance for further exploring the factors that affect the development of LDD.

**Table 6:** Correlation coefficient matrix

	T2*	FF$_U$	FF$_L$	Lip1.24	Lip1.12	Lip0.8	H$_2$O	Lac
T2*	1	$-$0.311	$-$0.303	$-$0.404	$-$0.359	$-$0.465	0.376	$-$0.252
FF$_U$	$-$0.311	1	0.968	0.398	0.44	0.428	$-$0.233	0.273
FF$_L$	$-$0.303	0.968	1	0.32	0.407	0.435	$-$0.228	0.197
Lip1.24	$-$0.404	0.398	0.32	1	0.511	0.452	$-$0.274	0.497
Lip1.12	$-$0.359	0.44	0.407	0.511	1	0.372	$-$0.234	0.392
Lip0.8	$-$0.465	0.428	0.435	0.452	0.372	1	$-$0.146	0.26
H$_2$O	0.376	$-$0.233	$-$0.228	$-$0.274	$-$0.234	$-$0.146	1	$-$0.093
Lac	$-$0.252	0.273	0.197	0.497	0.392	0.26	$-$0.093	1

**Fig. 4:**
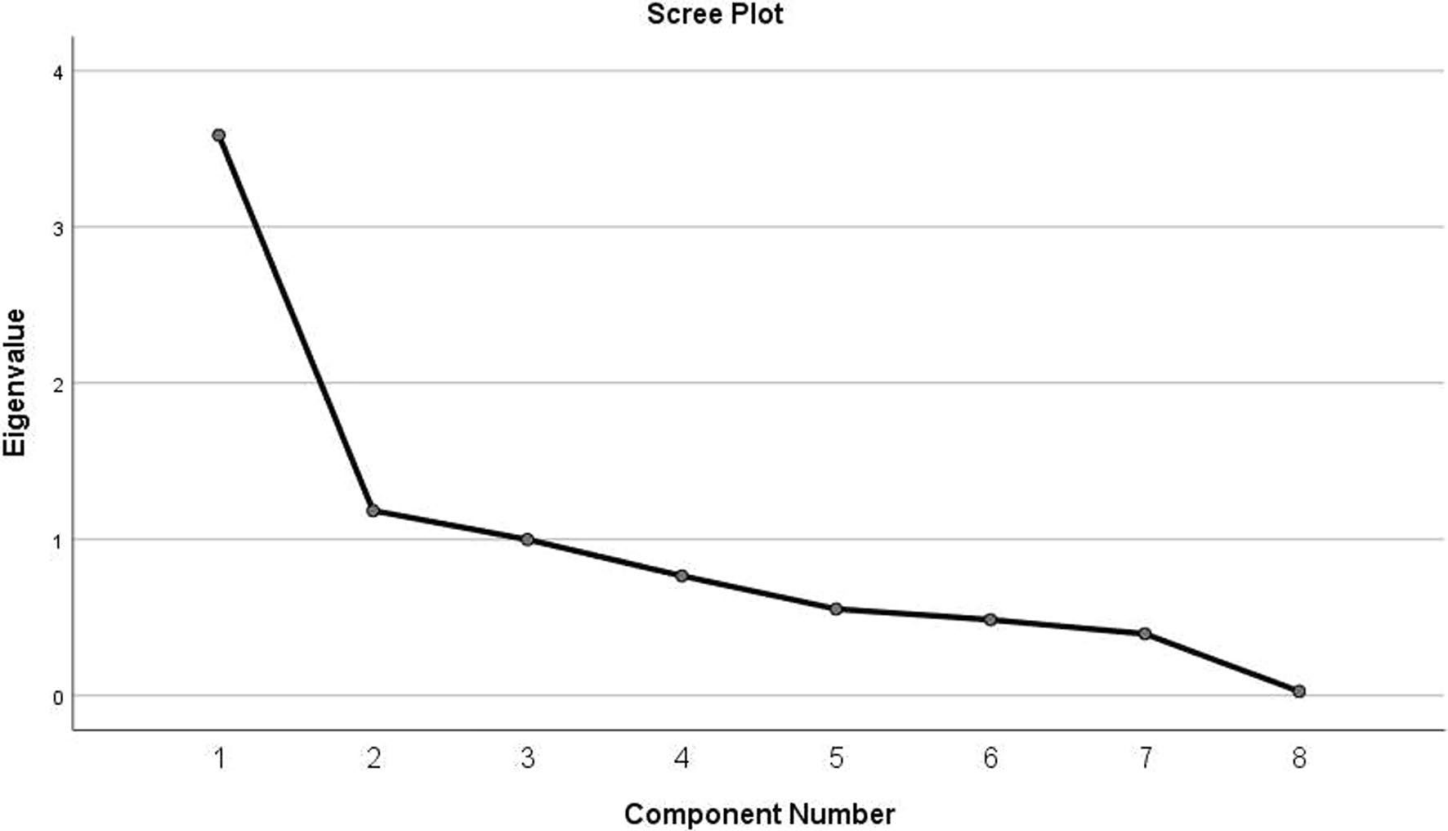
Scree plot

Table 7 shows that the feature values in order of size are 3.588, 1.185, 1.012, 0.766, 0.554, 0.485, 0.395, and 0.026. Based on the degree to which each principal component explained the variability of the data, we drew a scree plot (Fig. 4), which shows that the first three eigenvalues are greater than 1. Therefore, the first three common factors were selected for further study to better explain the contributions of the original variables. When more than three common factors were selected, the change in feature root values was relatively small, and the contribution to the original variable was also relatively small. Therefore, in subsequent analyses, we considered only the impact of the first three common factors on the original variable. The cumulative variance contribution rate of the first three common factors was 72.151% of the variance of the original variable. This result suggests that the selection of the first three common factors can maximize the information in the original variables and play a greater role in explaining the variance of the variables.

**Table 7:** Explanation of total variance

Component	Initial eigenvalues			Rotation sums of squared loadings		
	Total	% of Variance	Cumulative %	Total	% of Variance	Cumulative %
1	3.588	44.852	44.852	2.189	27.368	27.368
2	1.185	14.807	59.659	2.115	26.437	53.805
3	1.012	12.492	72.151	1.468	18.346	72.151
4	0.766	9.581	81.732	–	–	–
5	0.554	6.928	88.66	–	–	–
6	0.485	6.067	94.727	–	–	–
7	0.395	4.943	99.669	–	–	–
8	0.026	0.331	100	–	–	–

The rotated component matrix of the maximum variance method (Table 9) showed that the first common factor had a significant load on the FF values of the LBs adjacent to the LDs and was named the lumbar FF value factor. The second common factor had a significant load on the A$_{Lip}$ and A$_{Lac}$ values of the LBs and was named the lipid and lactate factor. The third common factor had a significant load on the A$_{H_2O}$ of the LBs and the T2* of the LDs and was named the water factor. Compared to the component matrix before rotation (Table 8), the meanings of the factors in the rotated component matrix were clearer and more reasonable, which was more conducive to the interpretation and application of subsequent data.

**Table 8:** Component matrix

MRI quantitative indicator	Component		
	1	2	3
FF$_U$	0.802	0.555	0.08
FF$_L$	0.767	0.619	0.033
Lip1.24	0.723	− 0.388	0.15
Lip1.12	0.705	− 0.175	0.155
Lip0.8	0.681	− 0.036	0.027
T2*	-0.634	0.281	0.402
Lac	0.534	− 0.454	0.47
H$_2$O	-0.43	0.163	0.758

**Table 9:** Rotated component matrix

MRI quantitative indicator	Component		
	1	2	3
FF$_L$	0.968	0.138	$-$0.126
FF$_U$	0.946	0.221	$-$0.117
Lac	0.037	0.841	0.061
Lip1.24	0.186	0.765	$-$0.278
Lip1.12	0.335	0.63	$-$0.208
Lip0.8	0.415	0.468	$-$0.272
H$_2$O	$-$0.097	$-$0.001	0.874
T2*	$-$0.166	$-$0.373	0.69

Through factor analysis and factor rotation, we obtained three main factors. The first common factor was the FF values of the LBs adjacent to the LDs, which represents changes in the distribution of LB fat in LDD patients. The second common factor was composed of the A$_{Lip}$ and A$_{Lac}$ of the LBs, which represents energy metabolism. The third common factor was composed of the A$_{H_2O}$ value of LB and the T2* value of LD, which represents water balance.

#### Factor score

As shown in the component score coefficient matrix (Table 10), the expressions for the FF value factor, lipid and lactate factor, and water factor of LB can be obtained by the following equation:

$$\begin{aligned} \mathrm{{F}}1= & {} 0.097 \times \left( {\mathrm{{T}}2\mathrm{{*}}} \right) + 0.506 \times \left( {\mathrm{{F}}{\mathrm{{F}}_\mathrm{{U}}}} \right) + 0.537 \times \left( {\mathrm{{F}}{\mathrm{{F}}_\mathrm{{L}}}} \right) - 0.106 \times \left( {\mathrm{{Lip}}1.24} \right) \\{} & {} + 0.027 \times \left( {\mathrm{{Lip}}1.12} \right) + 0.102 \times \left( {\mathrm{{Lip}}0.8} \right) + 0.084 \times \left( {{\mathrm{{H}}_2}\mathrm{{O}}} \right) - 0.158 \times \left( {\mathrm{{Lac}}} \right) , \\ \mathrm{{F}}2= & {} - 0.055 \times \left( {\mathrm{{T}}2\mathrm{{*}}} \right) - 0.096 \times \left( {\mathrm{{F}}{\mathrm{{F}}_\mathrm{{U}}}} \right) - 0.157 \times \left( {\mathrm{{F}}{\mathrm{{F}}_\mathrm{{L}}}} \right) + 0.396 \times \left( {\mathrm{{Lip}}1.24} \right) \\{} & {} + 0.289 \times \left( {\mathrm{{Lip}}1.12} \right) + 0.152 \times \left( {\mathrm{{Lip}}0.8} \right) + 0.212 \times \left( {{\mathrm{{H}}_2}\mathrm{{O}}} \right) + 0.554 \times \left( {\mathrm{{Lac}}} \right) , \\ \mathrm{{F}}3= & {} 0.486 \times \left( {\mathrm{{T}}2\mathrm{{*}}} \right) + 0.102 \times \left( {\mathrm{{F}}{\mathrm{{F}}_\mathrm{{U}}}} \right) + 0.08 \times \left( {\mathrm{{F}}{\mathrm{{F}}_\mathrm{{L}}}} \right) - 0.043 \times \left( {\mathrm{{Lip}}1.24} \right) \\{} & {} + 0.012 \times \left( {\mathrm{{Lip}}1.12} \right) - 0.065 \times \left( {\mathrm{{Lip}}0.8} \right) + 0.738 \times \left( {{\mathrm{{H}}_2}\mathrm{{O}}} \right) + 0.242 \times \left( {\mathrm{{Lac}}} \right) . \end{aligned}$$

Using the total variance explanation in Table 7, we can obtain the comprehensive score with the following equation:

$$\begin{aligned} \mathrm{{F}}= & {} \frac{{27.368}}{{72.151}} \times \mathrm{{Value\ of\ Lumbar\ FF }} + \frac{{26.437}}{{72.151}} \times \mathrm{{Lipids\ and\ Lactic\ Acid\ Factors}} \\{} & {} - \frac{{18.346}}{{72.151}} \times \mathrm{{Water\ Factor}}. \end{aligned}$$

**Table 10:** Component score coefficient matrix

MRI quantitative indicator	Component		
	1	2	3
T2*	0.097	$-$0.055	0.486
FFU	0.506	$-$0.096	0.102
FFL	0.537	$-$0.157	0.08
Lip1.24	$-$0.106	0.396	$-$0.043
Lip1.12	0.027	0.289	0.012
Lip0.8	0.102	0.152	$-$0.065
H$_2$O	0.084	0.212	0.738
Lac	$-$0.158	0.554	0.242

This equation can be used to calculate the comprehensive score in factor analysis, where the common factor coefficient is divided by the rotated variance contribution rate and the total variance contribution rate. Because the influence of the water factor on LDD grade is opposite to that of the other two common factors, a negative sign is added before it. The comprehensive scores were grouped, and the number of samples with LDD grades I to V in each group was summarized, resulting in the distribution table of comprehensive scores for LDD grades (Table 11).

The comprehensive score table of the LDD grade divides the comprehensive scores S of each sample into five categories: S<$-$0.5, $-$0.5$\le$S<0, 0$\le$S<0.5, 0.5$\le$S<1, and S$\ge$1. As shown in Table 11, the LDD grades of samples with a comprehensive score greater than 1 were mainly Grade V, while the LDD grades of samples with a comprehensive score less than $-$0.5 were mainly Grades I, II, and III. As the overall score decreased, the number of samples with low LDD grades (grades I and II) showed an increasing trend. Taking samples with LDD grade I as an example, the comprehensive scores of these samples were all less than 0. There were 2 samples with comprehensive scores within the range of [$-$0.5, 0) and 6 samples with comprehensive scores less than $-$0.5. As the comprehensive score decreased, the number of samples with LDD grade I gradually increased. Therefore, calculating the comprehensive LD score provides a new method for diagnostic doctors to discover early LDD patients.

**Table 11:** Comprehensive score distribution of LDD grade

LDD grade			Comprehensive score			
	S<-0.5	-0.5$\le$S<0	0$\le$S<0.5	0.5$\le$S<1	S$\ge$1	Total
I	6	2	0	0	0	8
II	6	3	1	0	0	10
III	6	11	8	1	0	26
IV	1	9	10	2	1	23
V	1	4	9	10	5	29
Total	20	29	28	13	6	96

## Discussion

Research has shown that the degeneration of IDs is related to factors such as human ageing, mechanical stress, genetics, and nutritional disorders [12]. Among these factors, the continuous reduction in nutrition and long-term loss of nutrients are the key factors causing changes in ID cells [3, 13]. There are two main nutritional pathways for IDs, namely, the CEP pathway and the NF pathway. The CEP is closely related to the longitudinal growth of the vertebral body. Nutrients from the blood vessels in the vertebral body can diffuse to the ID through the interfaces among the bone marrow cavity, blood sinuses, and cartilage endplate, providing nutrients for the ID. This process of nutrient diffusion is crucial for the growth, repair, and maintenance of the spinal structure. Therefore, the CEP nutrient pathway is the main pathway for ID nutrient supply and plays a crucial role in maintaining the normal physiological function of the spine [14]. The histological origin of the CEP is consistent with that of vertebral body histology. When the metabolism of nutrients in the vertebral body is disrupted or the supply is reduced, the nutritional supply function of the CEP will inevitably be damaged, ultimately leading to ID degeneration. Due to the direct impact of the vertebral body on the lifespan and characteristics of LDs, using specific vertebral MRI quantitative indicators for targeted and specific detection and analysis is an effective method. By observing the changes in quantitative indicators of vertebral MR images under pathological conditions and correlating them with the degree of LDD, as evaluated using the Pfirrmann grading method, it is possible to better understand the upstream mechanism of LDD.

In recent years, MRI quantitative technology has provided a powerful tool for the complete quantitative evaluation of LDD by utilizing digital calculations and MRI image analysis. This technology covers multiple biological metabolic indicators. [15] found significant differences in N-acetyl/carbohydrate, N-acetyl/lipid, and lactate regions among different Pfirrmann grades of LDD in cadavers through the Tukey-Kramer test, as well as significant differences in N-acetyl/choline, N-acetyl/lipid, and lactate regions before and after bovine LD denaturation. [16] used gas chromatography-mass spectrometry (GC-MS) to analyse changes in plasma metabolic levels in LD patients. Multivariate statistical analysis and metabolic network analysis revealed that the main upregulated metabolites were glutamate, aspartic acid, and glycine, while the downregulated metabolite was glucose 1-phosphate. [17] used H-HR-MAS NMR to determine the metabolite concentration in LDs and evaluated the correlation between metabolite concentration and LD with different Pfirrmann grades. The spectral analysis of LDs with Pfirrmann grades IV and V revealed that the concentrations of creatine, glycine, hydroxyproline, alanine, leucine, valine, acetate, isoleucine $\alpha /\beta$, glucose and inositol were significantly greater, while the intensity of the N-acetyl peak of chondroitin sulfate was decreased.

In contrast to previous studies, this study analysed metabolites, including Lip, Lac, N-acetyl, Cho, and H_2_O, of the vertebral body, which, as the upstream nutrient source, may be related to LDD. In previous studies, biochemical metabolites in the intervertebral disc were often analysed to determine the outcomes of LDD changes. This study innovatively analysed the upstream nutrient supply side of the disc, so the results more closely reflect the interrelation between the nutritional status of the LB and LD performance. Due to the limited vertebral display of N-acetyl and Cho, which may be limited by the MRS resolution of the MRI instrument, these two metabolic indicators were not analysed. During the LDD process, these MRI quantitative indicators typically exhibit specific changes [18], but their usefulness in evaluating the LDD process is not clear and has not been reported in the literature.

In this study, we used a factor analysis method to identify MRI quantitative indicators with strong correlations by observing the interrelationships between the measured MRI quantitative indicators in the LBs and LDs, i.e., the A$_{Lip}$, A$_{H_2O}$, A$_{Lac}$, and FF values of the LB and the T2* values of the LD, and extracting a small number of important interpretable common factors. Factor analysis is a mature and classic statistical method that can identify potential correlations between multiple variables, providing clues for further research. This method can compress multiple related variables into fewer potential factors through dimensionality reduction methods, thereby reducing the complexity of data analysis. These common factors can serve as covariates for the BP neural network LDD grade diagnostic model, helping to reduce model complexity and make the model more intuitive and easier to interpret. In addition, the comprehensive scores of each LD can be calculated through common factors, and the LDD grade can also be analysed based on the comprehensive scores.

The results of this study show that the first common factor is composed of the lumbar FF values measured in the upper and lower vertebral bodies of the LDs. Because FF values can be used to reflect the distribution of fat in tissues, this factor may represent changes in the content of fat in the LBs in LDD patients. Reflecting the health status of intervertebral discs is closely related to quantifying the vertebral fat content.

The second common factor is composed of the A$_{Lip}$ and A$_{Lac}$ in the LBs, which may represent the energy metabolism status of LDD patients. The lipid peak represents the abundance of lipids, while the lactate peak reflects the accumulation of lactic acid in the body, which may be caused by disorders in energy metabolism. Therefore, this factor may reflect the dual changes in lipid metabolism and energy metabolism in LDD patients.

The third common factor is composed of the A$_{H_2O}$ value of the LB and the T2* value of the LD, which are closely related to the water content, so this factor reflects the water balance status of LDD patients. Water balance plays an important role in maintaining the normal structure and function of intervertebral discs, thus providing valuable information for studying the mechanism and treatment of LDD.

These three common factors provide valuable information for LDD research and reveal some key indicators. By analysing these factors in depth, we can better understand the causes of LDD and provide a basis for diagnosis and treatment. Therefore, these three common factors can serve as covariates for evaluating LDD diagnostic models, optimizing the accuracy and reliability of these models, and play a crucial role in the early diagnosis and treatment of LDD.

In summary, this study provides new approaches and methods for the diagnosis and treatment of LDD, as well as a new perspective for the application of MRI technology, which has important implications and reference value for clinical practice. In response to the problems encountered during the data collection process, areas for improvement can be considered in subsequent studies, such as the following: 1) Ensuring the accuracy and consistency of data collection. We plan to develop a more detailed and specific data collection plan, focussing on the reliability of data sources and protecting personal privacy and data security during the collection process, and set up data monitoring points to check data error rates and inconsistencies. 2) Reducing the data loss rate. When collecting quantitative MRI data from patients, attention should be given to the issue of data loss. Measures should be taken to reduce the data loss rate, such as imaginative navigation, fat suppression, and Laplace filtering, to increase the dimensionality and accuracy of the data and ensure the authenticity and reliability of the results.

Future work can also be carried out in the following areas: 1) Expanding the sample size of the dataset. The collection of more patient data can increase the detection of LDD indicators, increase the understanding of influencing factors, and reduce data bias. 2) The exploration of advanced machine learning and omics analysis techniques, such as deep learning, one-dimensional convolutional neural networks (1D-CNNs), and support vector machines (SVMs), can assist in establishing more accurate, comprehensive, and fast LDD diagnostic models, enhancing clinical application value. 3) Exploring other research methods, such as magnetic resonance elastography (MRE) and magnetic resonance spectroscopy (MRS), can deepen the understanding of LDD levels when used in conjunction with MRI technology.

## Data Availability

All data generated during the project will be made freely available via 909th Hospital (Affiliated Southeast Hospital, Xiamen University). DOIs to these data will be provided (as part of the DataCite program) and cited in any published articles using these data and any other data generated in the project. There are no security, licensing, or ethical issues related to these data.
